# Fibromatosis in vertical rectus abdominis myocutaneous flap imitating tumor recurrence after surgery for locally advanced rectal cancer: case report

**DOI:** 10.1186/s12957-016-0818-4

**Published:** 2016-03-03

**Authors:** Mariusz Adam Goscinski, Knut Håkon Hole, Elin Tønne, Truls Ryder, Krystyna Kotanska Grøholt, Kjersti Flatmark

**Affiliations:** Departments of Gastroenterological Surgery, The Norwegian Radium Hospital, Oslo University Hospital, Oslo, Norway; Departments of Pathology, The Norwegian Radium Hospital, Oslo University Hospital, Oslo, Norway; Departments of Radiology and Nuclear Medicine, The Norwegian Radium Hospital, Oslo University Hospital, Oslo, Norway; Department of Medical Genetics, The Norwegian Radium Hospital, Oslo University Hospital, Oslo, Norway; Faculty of Medicine, Institute for Clinical Medicine, University of Oslo, Oslo, Norway

**Keywords:** Locally advanced rectal cancer, Familial adenomatous polyposis, Abdominoperineal excision, Vertical rectus abdominis myocutaneous flap, Fibromatosis, *APC*

## Abstract

**Background:**

Abdominoperineal excision is performed in patients with locally advanced, low rectal carcinoma. Reconstruction of the dorsal vagina and perineum using the vertical rectus abdominis myocutaneous flap following extensive surgery results in favorable surgical outcome and quality of life. However, the rectus abdominis muscle, as part of the anterior abdominal wall, may develop fibrous lesions also as a transplant.

**Case presentation:**

A 39-year-old female patient with low rectal cancer and extensive colorectal polyposis was treated with neoadjuvant chemoradiotherapy followed by colectomy and abdominoperineal excision with resection of the dorsal vaginal wall and subsequent reconstruction of the perineum using the vertical rectus abdominis myocutaneous flap. At the 6-month follow-up, a suspected 2 × 2 cm tumor recurrence was detected in the transposed tissue and was subsequently surgically removed. Histologic examination concluded with fibromatosis. Genetic testing revealed a known disease-causing mutation in the adenomatous polyposis coli gene, confirming the diagnosis of familial adenomatous polyposis.

**Conclusions:**

Fibromatosis may affect the anterior abdominal wall, that is the rectus abdominis muscle, at the primary site or may develop in the muscle after its transposition into the perineum at pelvic reconstruction. Fibromatosis in the muscle flap after pelvic reconstruction may present a difficult diagnostic challenge for the multidisciplinary team.

## Background

For low rectal cancer, abdominoperineal excision (APE) is usually the procedure of choice, and in locally advanced cases, the majority of patients require extralevatoric excision to achieve adequate circumferential resection margins [1]. Tumor involvement of the posterior vaginal wall implicates not only the necessity for resection but also the transposition of tissue to reconstruct the vaginal continuity and promote wound healing [2, 3]. Commonly, a vertical rectus abdominis myocutaneous (VRAM) flap is used for this purpose, as it has been shown to decrease wound complications after surgery [4, 5].

## Case presentation

A 39-year-old, healthy, non-smoking female patient with no family history of cancer was referred on suspicion of rectal cancer. Digital rectal examination and subsequent rigid proctoscopy revealed a low rectal tumor and multiple polyps in the rectum. Subsequent colonoscopy showed extensive polyposis of the entire colon. The tumor biopsy confirmed the presence of a rectal adenocarcinoma, while dysplastic changes were found in the polyp biopsies. No distant metastases were detected on the thoracoabdominal computed tomography (CT) scan, and baseline carcinoembryonic antigen (CEA) was 8 μg/L. Pelvic magnetic resonance imaging (MRI) revealed a locally advanced rectal cancer with extra-mesorectal growth into the rectovaginal septum and the lower posterior vaginal wall as well as a suspected growth into the anterior aspect of the coccygeal bone with multiple suspected malignant lymph nodes within the mesorectum (T4N2). Upon evaluation by our multidisciplinary team (MDT), she was scheduled for neoadjuvant chemoradiotherapy (CRT) followed by surgery. Neoadjuvant treatment was given as CT planned radiotherapy (daily 2-Gy fractions, 5 days per week; the initial 23 fractions to the macroscopic tumor and areas at risk, and the two final fractions adapted to the macroscopic tumor) with concomitant capecitabine (825 mg/m^2^) on days of RT. The response evaluation showed excellent tumor volume response, but with similar organ involvement as at baseline, and she proceeded to surgery 8 weeks after CRT completion. Surgery involved total colectomy, APE with resection of the posterior vaginal wall and coccygeal bone with subsequent reconstruction of the perineum with right-sided VRAM flap, and a terminal ileostomy. After an uneventful recovery, she was discharged 15 days postoperatively. Histologic examination of the specimen showed a 35-mm large adenosquamous carcinoma removed with free resection margins and metastasis in one of four local lymph nodes (pT2N1). In addition, multiple dysplastic adenomas were present in the entire colon.

**Fig. 1:**
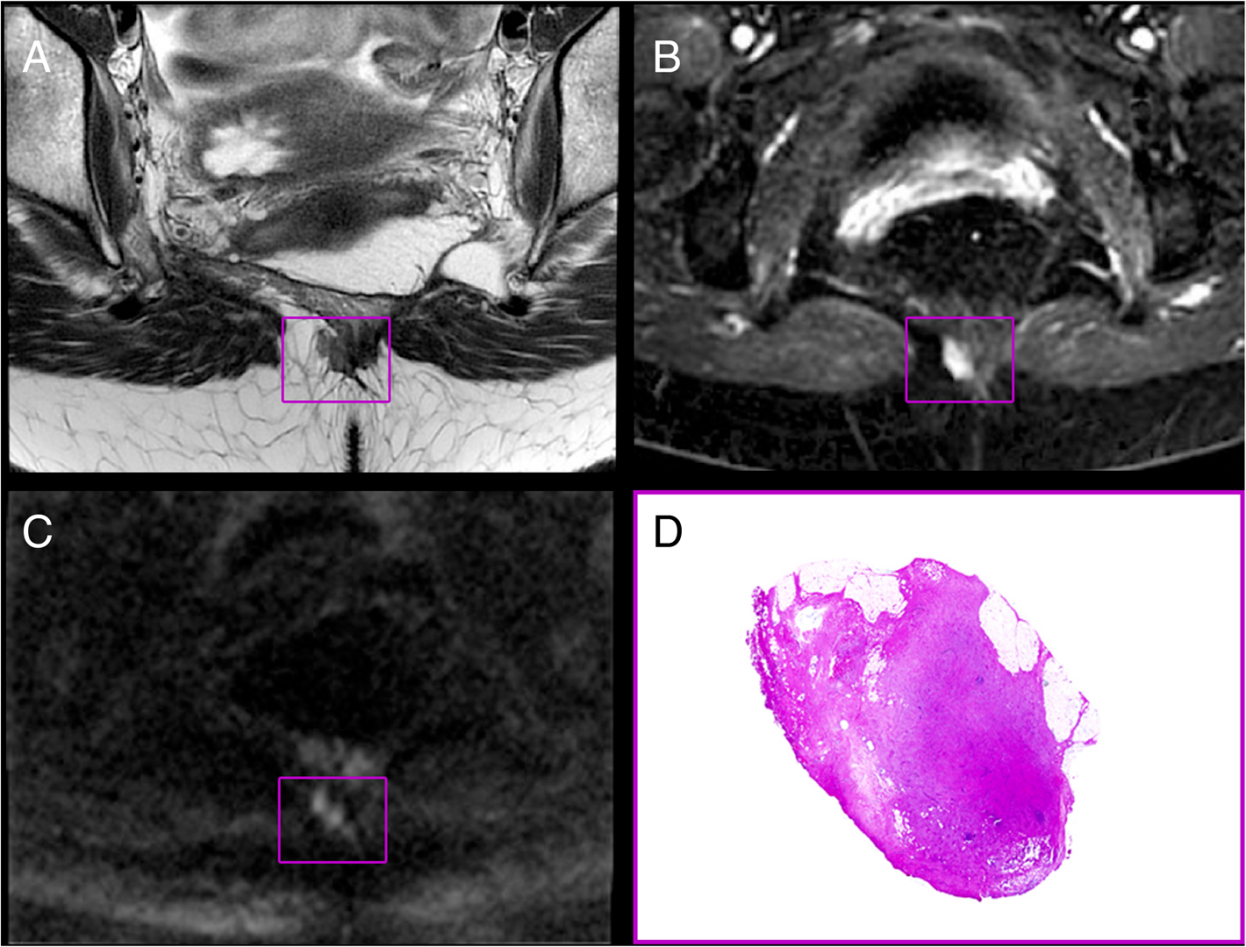
Transversal MR images (**a**–**c**) and hematoxylin and eosin stained tumor section (**d**); localization outlined in *purple squares*. **a** T2-weighted image. **b** Dynamic contrast enhanced parameter map (K^TRANS^). **c** Heavily diffusion weighted image (b1500). MRI-indicated and rich vascularization (**b**) and high cellularity (**c**)

**Fig. 2:**
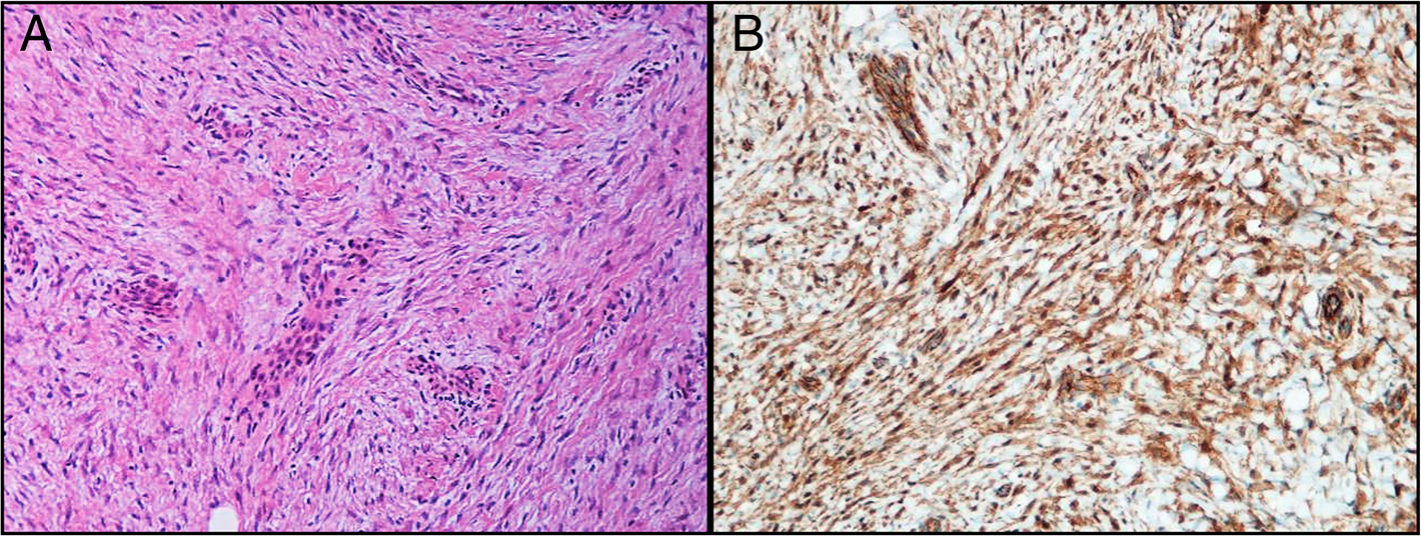
Histologic high power views of the resected tumor. **a** Hematoxylin and eosin staining. **b** ß-catenin immunohistochemical staining

At the routine follow-up 6 months postoperatively, clinical examination revealed a 2 × 2 cm tumor located in the muscle tissue of the VRAM flap, and an early local recurrence was suspected. Pelvic MRI showed a 10 × 16 mm rich vascularized and cell dense lesion corresponding to the clinically detected tumor (Fig. 1). No other pathological findings were made on CT or MRI, and CEA was 1 μg/L. The lesion was surgically excised with a wide local resection of the tumor. Interestingly, histologic examination of the removed lesion described a mass composed of fatty tissue, fibrocytes, and fibroblasts arranged in broad, sweeping fascicles infiltrating the adjacent striated muscle tissue. No dysplasia was observed, but mitoses were present. Immunohistochemistry showed positive staining for β-catenin (ABCAM, Cambridge, USA), and the tumor was diagnosed as a fibromatosis (Fig. 2). The medical geneticists suspected familial adenomatous polyposis (FAP), and testing of the *APC* gene, revealed a known disease-causing mutation c.3317dupG (p.Ala1107Serfs*12), confirming this diagnosis.

## Conclusions

Deep fibromatosis (desmoid tumor) is a benign, myofibroblastic proliferation that rarely occurs in the general population but frequently in one of the hereditary cancer predisposition conditions known as FAP or a variant of FAP called Gardner syndrome [6, 7]. Nearly all deep fibromatoses have somatic β-catenin or *APC* gene mutations leading to intranuclear accumulation of β-catenin [8]. Nuclear detection of β-catenin by immunohistochemistry is reported in 80–98 % of cases and is used in diagnostics [9, 10]. Desmoid tumors in FAP are typically associated with mutations in the *APC* gene located downstream of codon 1400 [11]. However, the genotype-phenotype association is not absolute, as in this case, where the mutation is approximately 300 codons upstream of codon 1400. The clinical presentation of fibromatosis is variable, depending on the location and the extent of the lesion. Desmoids may occur in any musculo-aponeurotic tissue structures of the body as well as within the abdominal cavity. Extra-abdominal fibromatoses arise typically from the rectus or internal oblique muscles and fascia predominantly in young women. Previous surgery and trauma sites and irradiated tissues may also be a characteristic place of desmoid expansion [12, 13].

Taking into consideration all of these factors, our patient was at high risk of developing fibromatosis. However, as the standard follow-up procedure for patients undergoing rectal cancer treatment is to focus on early detection of recurrence and metastasis, and furthermore considering the patient’s primary diagnosis, the MRI findings, the lesion site, and its rapid development, the initial conclusion was local recurrence. Fibromatosis as a differential diagnosis was thus not considered, and the MDT opted for direct surgical removal, as the lesion’s location and size were found to be appropriate for primary surgical removal. The use of PET-CT was discussed, but high-quality CT and MRI analysis did not reveal additional suspected malignant lesions and were considered sufficient for making treatment decisions. The histological examination, however, revealed fibromatosis in the VRAM flap in the perineum instead of the expected recurrence. It is noteworthy that no sign of fibromatosis in the abdominal rectus muscle was found on the CT prior to the primary surgery.

Currently, the universal approach to management of desmoid tumors (both abdominal and extra-abdominal) favors the “watch and wait” approach rather than surgical removal. This is based on the observation that size stabilization or spontaneous regression may occur while surgery in itself may trigger the growth of new lesions [14, 15].

In this case, no histological evidence (biopsy) was available to support such a decision, nor was the genetic evaluation implemented. The lesion was considered to be a suspected local relapse without distant metastases at the time of diagnosis and was dealt with according to national guidelines for treatment of recurrent rectal cancer.

In this case, fibromatosis in transposed rectus abdominis muscle tissue imitated a rectal cancer recurrence. Although rare, but with pelvic reconstructive surgery becoming more frequent, this may be a relevant differential diagnosis for the MDT to consider, particularly in patients with FAP.

## Consent

Written informed consent was obtained from the patient for publication of the case report and any accompanying images. A copy of the written consent is available for review at the editor of this journal.

## Abbreviations

APC: adenomatous polyposis coli
APE: abdominoperineal excision
CEA: carcinoembryonic antigen
CRT: neoadjuvant chemoradiotherapy
CT: computed tomography
FAP: familial adenomatous polyposis
Gy: gray
MDT: multidisciplinary team
MRI: magnetic resonance imaging
N: lymph nodes
T: tumor
VRAM: vertical rectus abdominis myocutaneous
